# Brain Connectivity Plasticity in the Motor Network after Ischemic Stroke

**DOI:** 10.1155/2013/924192

**Published:** 2013-04-24

**Authors:** Lin Jiang, Huijuan Xu, Chunshui Yu

**Affiliations:** ^1^Department of Radiology, Tianjin Medical University General Hospital, Tianjin 300052, China; ^2^School of Medical Imaging, Tianjin Medical University, Tianjin 300070, China

## Abstract

The motor function is controlled by the motor system that comprises a series of cortical and subcortical areas interacting via anatomical connections. The motor function will be disturbed when the stroke lesion impairs either any of these areas or their connections. More and more evidence indicates that the reorganization of the motor network including both areas and their anatomical and functional connectivity might contribute to the motor recovery after stroke. Here, we review recent studies employing models of anatomical, functional, and effective connectivity on neuroimaging data to investigate how ischemic stroke influences the connectivity of motor areas and how changes in connectivity relate to impaired function and functional recovery. We suggest that connectivity changes constitute an important pathophysiological aspect of motor impairment after stroke and important mechanisms of motor recovery. We also demonstrate that therapeutic interventions may facilitate motor recovery after stroke by modulating the connectivity among the motor areas. In conclusion, connectivity analyses improved our understanding of the mechanisms of motor recovery after stroke and may help to design hypothesis-driven treatment strategies and sensitive measures for outcome prediction in stroke patients.

## 1. Introduction

Motor disability is the most common deficit after ischemic stroke. Following initial damage, stroke patients can usually recover to some extent, which may be related to structural and functional modifications in surviving brain tissue. Studies on stroke rats and patients have revealed that spontaneous recovery of the motor function after stroke is associated with brain plasticity [1–5]. Neuroimaging techniques, especially the multimodality MRIs, have significantly contributed to our understanding of the mechanisms of stroke recovery by characterizing brain structural and functional changes after stroke [6–10]. Electroencephalogram (EEG) has high temporal resolution; however, it exhibits low spatial resolution and cannot record signals from deep brain tissues. Positron emission tomography (PET) is a radioactive technique with a relatively low spatial resolution. However, MRI is a noninvasive technique with high spatial resolution and is especially suitable for longitudinal connectivity studies. Moreover, MRI is a multimodality imaging technique that can be used to investigate both anatomical and functional connectivities. Structural MRI studies have revealed extensive atrophy in brain regions that connect with stroke lesions. More importantly, increased grey matter volume and cortical thickness were also found in the motor-related areas and hippocampus either during spontaneous recovery or after treatments [11–13]. Task-based functional MRI (fMRI) or PET has been extensively used to investigate brain activation changes during stroke recovery [14–16] and has provided important information on the patterns of functional reorganization after stroke [14–19]. Movements of the stroke-affected hand are initially activated extensive brain regions in stroke patients [16, 20–22] and rats [6, 23–30]. However, this initial widespread activation does not predict good functional recovery [31–33], whereas normalization of the activation pattern to prestroke level is associated with good outcome [34].

Knowing brain structural or functional alteration in a particular region does not tell us how this region interacts with other regions, which modulates behavior in concert [35]. Recently, connectivity-based methods have been used in stroke patients and rats to demonstrate anatomical and functional connectivity changes after stroke; these changes may be related to clinical deficits and functional recovery. These methods may provide great insight into network dysfunction [21, 36–39] and functional reorganization [40, 41] from a system perspective; they will also contribute to predict outcomes and to develop new therapeutic interventions [40, 41]. In this review, we focused on recent neuroimaging studies employing connectivity-based analyses to investigate connectivity changes in the motor network after ischemic stroke.

## 2. Neuroimaging Methods for Brain Connectivity

Connectivity models are based on the concept that the brain is organized by segregation of specialized and anatomically distinct brain regions which are functionally integrated in a network mediating motor, sensory, or cognitive processing [42]. There are four common connectivity models: the anatomical connectivity, functional connectivity, effective connectivity, and network models.

### 2.1. Anatomical Connectivity

Diffusion tensor imaging (DTI) is the most common method that can detect changes in anatomical connectivity *in vivo* [43]. In normal white matter, water molecules move relatively freely in a direction parallel to fiber tracts in contrast to the restricted movement across the tracts; this phenomenon is referred to as diffusion anisotropy. Based on diffusion anisotropy, the white matter fiber tracts can be reconstructed using diffusion tensor tractography (DTT) [44, 45]. Anatomical connectivity can be assessed by either visualizing fiber tracts or evaluating diffusion characteristics. Fractional anisotropy (FA) and mean diffusivity (MD) are the most commonly used DTI measures; MD reflects the average diffusion amplitude, and FA represents the degree of directionality of microstructures, such as axons, myelin, and microtubules [44, 46]. DTI measures can be analyzed by either data-driven methods (voxel-based analysis and tract-based spatial statistics) or hypothesis-driven methods (region of interest (ROI) analysis and tractography-based analysis). DTI and DTT have been used in the evaluation of the white matter damage and reorganization in stroke patients [47–51]. In different poststroke stages, alterations of DTI measures represent different pathological processes: MD decreases at acute stage represent cell swelling; MD increases and FA decreases at subacute stage denote cell lysis and demyelination; and FA increases at chronic stage suggests axonal regeneration or remyelination [52–55]. 

It is well recognized that diffusion-weighted imaging (DWI) is capable of yielding considerably more information than that contained in the diffusion metrics derived from DTI due to the fact that DTI is based upon a Gaussian approximation of the diffusion displacement probability function. However, non-Gaussian diffusion in the brain really exists [56, 57] and is believed to arise from diffusion restricted by barriers, such as cell membranes and organelles, as well as the presence of distinct water compartments [58, 59]. Diffusional kurtosis imaging (DKI) has been proposed to quantify non-Gaussian diffusion through the estimation of the diffusional kurtosis [60–62]. The mean kurtosis (MK), a principal metric of the diffusional non-Gaussianity, is of potential interest to the study of white and gray matter integrity. DKI can provide additional information (non-Gaussian diffusion) that cannot be obtained from DTI, and it has been suggested that DKI may provide enhanced contrast between ischaemic and normal tissues [63]. Besides, MK is sensitive to hyperacute and acute stroke changes and exhibits more significant differences between ischaemic and normal tissues than measures (MD and FA) derived from DTI [64]. Thus, diffusional kurtosis is sensitive to diffusional heterogeneity and DKI may be a promising method in the assessment of ischemic stroke [61, 63, 64]. 

### 2.2. Functional Connectivity

Functional connectivity is defined as temporal correlation between spatially remote neurophysiological events. The resting-state functional connectivity (rsFC) measures temporal coherence of low-frequency fluctuations (<0.1 Hz) of the blood oxygenation level-dependent (BOLD) signals between spatially remote brain regions. Correlation of these signals with underlying neural activity indicates that these fluctuations are of functional significance [65–67]. Strong rsFC is first reported in motor network [68] and then is confirmed in other functional systems [69, 70]. The rsFC is present between brain regions with both monosynaptic and polysynaptic connections and depends on intact connections within a specific polysynaptic pathway [71]. It is a promising means of assessing intrinsic information transfer within a functional network while avoiding task-induced confounds [72].

A hypothesis-driven method has been extensively used to analyze rsFC changes. In this method, an ROI should be predefined based on hypotheses, and then the rsFC pattern of this ROI is obtained by computing correlations in the fMRI time courses between the ROI and every voxel of the whole brain [73]. The independent component analysis (ICA) is the most popular data-driven method to assess rsFC [74, 75]. It has been used to extract brain functional networks and to identify intranetwork rsFC changes [76]. The functional connectivity density (FCD) mapping is another data-driven method and measures the number of functional connections per voxel [77, 78]. These rsFC analytic methods are suited for the investigation of how multiple distributed networks are disrupted by stroke and are reorganized after stroke and what patterns of connectivity are most likely to be behaviorally relevant [40, 41].

### 2.3. Effective Connectivity

In contrast to the nondirectional, correlative nature of functional connectivity, effective connectivity measures the causal influence that one brain area exerts over another under the assumption of a given mechanistic model. This approach can provide crucial knowledge about the direction of information flow and ultimately what kinds of computations are being performed in the system by showing which nodes in a network are driven by which other nodes [35, 40, 41, 79].

Effective connectivity is based on mathematical models that are usually applied to task-based neuroimaging data. The psychophysiological interactions (PPI) model is a relatively simple method to estimate effective connectivity from neuroimaging data [80]. This exploratory method explains activity of a cortical area by means of an interaction term between the influence of another area and some experimental or psychological parameter [80, 81]. Granger causality analysis of effective connectivity is another exploratory method that identifies those voxels that are sources or targets of directed influence for any seed region [82]. In contrast to these exploratory methods, structural equation modelling (SEM) [83] and dynamic causal modeling (DCM) [84] are hypothesis-driven approaches that require a priori defined network of brain regions to estimate effective connectivity from neuroimaging data [81]. In addition, the effective connectivity analysis can also be used to resting-state fMRI data [82].

### 2.4. Network Model

In the framework of graph theory, the brain is described as a graph comprising a certain number of nodes (corresponding to brain regions) that are connected by edges (corresponding to anatomical connectivity, functional connectivity, effective connectivity, or other measures of interregional interactions) [85–88]. The network's structure can be assessed by measuring its clustering coefficient, a measure of segregation, that reflects the degree to which nodes are clustered, and the shortest path length, a measure of integration, that reflects the minimal number of edges between any pair of nodes. A high clustering coefficient and a low average shortest path length indicate a small-world network topology, which is proposed to be an optimal network configuration for global information transfer and local processing [85, 86, 88]. In addition to measures that assess the efficiency of the whole network, the node degree (i.e., the number of edges connected to a given node) and the betweenness centrality (i.e., the fraction of the shortest paths that pass through a given node) are used to assess the importance of a given node. Brain regions featuring a high node degree and a high centrality are assumed to serve as “hubs” mediating functional integration between regions. 

## 3. Brain Connectivity Changes After Stroke

Brain reorganization after stroke is a dynamic process, which considerably differs across patients, depending on lesion location, time since stroke, severity of motor impairment, premorbid state, and even genetics. These contributing factors make it unlikely that one universal measure exists which is suitable for all patients. Consequently, a variety of connectivity analyses have been performed to investigate motor recovery mechanisms after ischemic stroke.

### 3.1. Changes in Anatomical Connectivity After Stroke

DTI and DTT are powerful methods to detect not only impairments but also modifications in anatomical connectivity after stroke [89, 90]. The motor recovery mechanisms in ischemic stroke patients have been summarized as (1) recovery of a damaged lateral corticospinal tract (CST); (2) subcortical perilesional reorganization; (3) ipsilateral motor pathway from the unaffected motor cortex to the affected extremities; and (4) collateral pathway of the pyramidal tract [89].

#### 3.1.1. Recovery of a Damaged Lateral CST

Pannek et al. [91] investigated longitudinal CST white matter connectivity changes during stroke recovery using probabilistic DTT in 10 patients with cerebral infarcts. They found that the anatomical connectivity of the CST at the cortical regions within the affected hemisphere was enhanced over time and that the enhanced connectivity was correlated with stroke recovery. Schaechter et al. [92] examined the relationship between microstructural status of brain white matter tracts and motor skill of the stroke-affected hand in patients with chronic stroke. They found that motor skill significantly and positively correlated with FA values of the ipsilesional and contralesional CST in these patients. They also found that patients with better motor skill had elevated FA of the bilateral CSTs compared to controls. These findings may support the hypothesis of recovery of the damaged lateral CST. However, our previous study of dynamic evolution of the diffusion indices in the degenerated CST did not show any significant plastic changes after ischemic stroke although a slightly elevated FA was found 1 year after stroke [93]. The discrepancy between these studies may be ascribed to methodological differences because we only studied the CST section at the level of pons, which may miss the portion of the CST that exhibits plastic changes.

#### 3.1.2. Subcortical Perilesional Reorganization

At subacute stage of stroke, decreased FA in ipsilesional white matter has been commonly reported, which reflects demyelination or axonal loss [8, 47, 94]. This initial decrease of FA may be chronically followed by normalization or elevation in the borderzone of the ischemic lesion [55], which could be further enhanced by treatments with neural progenitor cells [9], sildenafil [95], or erythropoietin [96]. Pathological examination confirmed a high density of axons and myelin in this region [9, 95]. The subcortical perilesional reorganization is also reported at the levels of the corona radiata and pons in stroke patients [97–101]. These findings suggest that rearrangement of white matter in the ischemic perilesional areas is accompanied by preservation or restoration of neuronal connectivity and may be used to predict motor outcome after stroke.

#### 3.1.3. The Ipsilateral Motor Pathway Reorganization

The ipsilateral motor pathway from the unaffected motor cortex to the affected extremities has been regarded as one of the recovery mechanisms of stroke [102–106]. However, this mechanism is not supported by other studies [107, 108]. The location and size of the lesions, the different recovery rates, and the analytic methods used in these studies may partly account for the discrepancy [104]. As reviewed by Jang [99], understanding the ipsilateral motor pathway mechanism is important because it is related to poor motor outcome and can be changed with time or manipulated by various rehabilitative interventions [102, 108–113]. 

#### 3.1.4. Collateral Pathway of the Pyramidal Tract

The pyramidal tract is known to possess collateral pathways in the human brain [114–116]. The aberrant pyramidal tract refers to the collateral pathway of the pyramidal tract through the medial lemniscus in the brainstem, which separates from the original pyramidal tract at the level of the midbrain and the pons and descends through the medial lemniscus [116]. Recently, several studies have suggested that the aberrant pyramidal tract may contribute to motor recovery in stroke [117–120]. Other motor-related pathways, including the corticorubrospinal and corticoreticulospinal tracts and the transcallosal motor pathways, may also contribute to the potential for functional recovery [121, 122]. For example, Rüber et al. reported increased white matter integrity in the corticorubrospinal tract within the vicinity of the red nuclei. They also found strong correlations between microstructural properties of this tract and the level of motor function in chronic stroke patients, which highlight a compensatory function of the corticorubrospinal system [122].

### 3.2. Changes in rsFC After Stroke

In a longitudinal rsFC study of rats after unilateral stroke [37], the authors found considerable loss of rsFC between the ipsilesional and contralesional primary sensorimotor cortex regions, alongside significant sensorimotor function deficits in the first days after stroke. The interhemispheric rsFC restored in the following weeks but remained significantly reduced up to 10 weeks after stroke in rats with lesions that comprised both the subcortical and cortical tissues. Intrahemispheric rsFC between the primary somatosensory and motor cortex areas was preserved in the lesion border zone and moderately enhanced contralesionally. The temporal pattern of changes in rsFC between the bilateral primary motor and somatosensory cortices correlated significantly with the evolution of sensorimotor function scores [37]. Subsequently, these authors confirmed that the decreased interhemispheric rsFC between the ipsilesional and contralesional primary sensorimotor cortex regions is associated with a decrease in transcallosal manganese transfer between these regions [38]. Using the same stroke rat model, these authors recently found that the degree of functional recovery after stroke was associated with the extent of preservation or restoration of the ipsilesional corticospinal tracts in combination with reinstatement of the interhemispheric neuronal signal synchronization and normalization of small-world cortical network organization [123]. 

Similar rsFC changes were also found in stroke patients. Immediately after stroke, significantly decreased rsFC of the ipsilesional primary sensorimotor cortex, especially the interhemispheric rsFC, was consistently reported [36, 124–126]. These decreased rsFCs were then gradually increased during recovery process and finally restored to the levels of near normal, normal, or above normal [36, 124–126]. In acute stroke patients, Carter and colleagues reported that disruption of the interhemispheric rsFC within the attention network was significantly correlated with abnormal detection of visual stimuli. In the somatomotor network, disruption of the interhemispheric rsFC was significantly correlated with upper extremity impairment. In contrast, intrahemispheric rsFCs within the normal or damaged hemispheres were not correlated with performance in either of the two networks [36]. This study emphasized the importance of the interhemispheric rsFC for specialized functions. Recently, a longitudinal study revealed that the rsFCs of the ipsilesional primary motor cortex (M1) with the contralesional thalamus, supplementary motor area (SMA), and middle frontal gyrus at onset were positively correlated with motor recovery at 6 months after stroke [125]. In subacute stroke patients, the interhemispheric rsFC was negatively correlated with the extent of corticospinal damage. Although corticospinal damage accounted for much of the variance in motor performance, the behavioral impact of rsFC was greater in subjects with mild or moderate corticospinal damage and less in those with severe corticospinal damage [40]. In chronic stroke patients, different outcomes are found to be associated with different change patterns of the rsFCs [127]. Moreover, the longitudinal changes of the rsFCs after stroke were correlated with modification of motor function [126]. 

Besides the altered rsFCs within the motor network after stroke, reduced interhemispheric connectivity was also found between the attention-related areas in stroke patients with neglect [36, 128] and the language areas in stroke patients with aphasia [129]. However, the effect of anatomical damage may extend beyond the lesioned area but remain within the bounds of the existing network connections. This concept is well elucidated by the finding of double dissociation of two cognitive control networks in patients with focal brain lesions. The degree of network damage correlates with a decrease in rsFC within that network while sparing the nonlesioned network [128]. 

### 3.3. Changes in Effective Connectivity After Stroke

Grefkes et al. applied dynamic causal modeling to investigate changes of effective connectivity among the M1, lateral premotor cortex (PMC), and SMA in subacute stroke patients when they performed visually paced hand movements with their left, right, or both hands [21]. Independently from hand movements, the intrahemispheric effective connectivity between the ipsilesional SMA and M1 and the interhemispheric effective connectivity of both the SMAs were significantly reduced. Furthermore, movements of the stroke-affected hand showed additional inhibitory influences from the contralesional to the ipsilesional M1 that correlated with the degree of motor impairment. For bimanual movements, interhemispheric communication between the ipsilesional SMA and contralesional M1 was significantly reduced, which was also correlated with impaired bimanual performance. The study suggested that the motor deficit of patients with a single subcortical lesion was associated with pathological interhemispheric interactions among the key motor areas. A dysfunction of effective connectivity between the ipsilesional and contralesional M1, and between the ipsilesional SMA and contralesional M1 underlied hand motor disability after stroke [21]. Changes in effective connectivity among the M1, SMA, and cerebellum (Ce) were also assessed in chronic stroke patients using dynamic causal modeling. Relative to healthy controls, stroke patients exhibited decreased intrinsic neural coupling between the M1 and Ce, but showed increased SMA to M1 and SMA to cerebellum couplings. The results demonstrate that connectivity alterations between motor areas may help to counterbalance a functionally abnormal M1 in chronic stroke patients [130].

The temporal evolution of intra- and interhemispheric effective connectivity was also investigated during motor recovery from the acute to the early chronic phase after stroke [131, 132]. Results showed reduced positive coupling of the ipsilesional SMA and PMC with the ipsilesional M1 in the acute stage. Coupling parameters among these areas increased with recovery and predicted a better outcome. Likewise, negative influences from the ipsilesional areas to the contralesional M1 were attenuated in the acute stage. In the subacute stage, the contralesional M1 exerted a positive influence on the ipsilesional M1. Negative influences from the ipsilesional areas on the contralesional M1 subsequently normalized, but patients with poorer outcome in the chronic stage now showed enhanced negative coupling from the contralesional upon the ipsilesional M1. These findings show that the reinstatement of effective connectivity in the ipsilesional hemisphere is an important feature of motor recovery after stroke. The shift of an early, supportive role of the contralesional M1 to enhanced inhibitory coupling might indicate maladaptive processes which could be a target of noninvasive brain stimulation techniques. Sharma et al. [133] investigated well-recovered stroke patients performing a motor imagery task, and found that the regional activations had returned to normal in the patients. In addition to reduced effective connectivity among the motor-related brain areas, they found significantly enhanced positive influences from the prefrontal cortex to both the SMA and PMC in stroke patients using an SEM analysis of effective connectivity. The authors suggest that enhanced coupling of the prefrontal areas might reflect the enhanced role of cognitive-related areas that facilitate movement planning to overcome the functional deficits caused by the damage to the motor pathways.

The effective connectivity can also be investigated during resting state. Using an SEM analysis, Inman and coauthors [134] focused on the intrinsic effective connectivity of top-down motor control in stroke patients exhibiting significant motor deficit. They found alterations in resting-state effective connectivity from the frontoparietal guidance systems to the motor network in stroke survivors. More specifically, diminished connectivity was found in connections from the superior parietal cortex to the M1 and SMA. These findings suggest that characterizing the deficits in resting-state connectivity of top-down processes in stroke survivors may help optimize cognitive and physical rehabilitation therapies by individually targeting specific neural pathway.

### 3.4. Changes in Network Efficiency After Stroke

The first preliminary study on the functional network efficiency changes after stroke was performed using graph theoretical measures on electroencephalography (EEG) data of 1 stroke patient and 8 healthy subjects when they performed a finger tapping task [135]. The authors found significant decrease in global and local efficiency in the patient's networks, reflecting a lower capacity to integrate communication between distant brain regions and a lower tendency to be modular. They also showed that these changes were associated with significant increases in the disconnected nodes and in the links of some other crucial vertices. The authors concluded that overall connectivity after stroke was governed by a lower number of brain regions in which increased connectivity could not compensate for the drastic reduction in information propagation.

The poststroke longitudinal changes in motor network efficiency have been reported in a resting-state fMRI study in which the authors used graph theory to assess changes in the topological configuration of the motor network from the acute phase to the chronic phase after subcortical stroke [126]. They found that over a year of recovery, motor execution network showed lower local efficiency within the network suggesting a shift towards a nonoptimal network configuration with less functional segregation. The overall decrease in network efficiency was paralleled by a stronger betweenness centrality of the ipsilesional M1 and cerebellum, the latter being a measure of the functional importance of a node for information processing. The increased importance of the ipsilesional M1 within the motor network after recovery was also indicated by stronger functional connectivity of this area with the contralesional motor areas [126]. Recently, the functional network characteristics of the whole brain were also investigated in stroke patients from the perspective of graph theory [136]. A longitudinal design was adopted in the study and the network measures were calculated based on the fMRI data when subjects were performing a finger tapping task. The authors showed that the brain networks shifted towards a nonoptimal topological configuration with low small worldness during the process of recovery. However, in an experimental stroke model, a rather different pattern of changes of network features were reported [123]. They found that the clustering coefficient, shortest path length, and small worldness of the motor network of stroke rats were significantly increased in the first poststroke days, and then these network measures declined towards a normal level. The authors speculate that the initial increase of network measures may be associated with initial excessive neuronal clustering and wiring, whereas the later decline toward a baseline small-world topology may be related to the refinement of the reorganized network. They also explain the discrepancy between their study and the findings of Wang et al. [126] by the differences between the (re)organization of rat and human brain and the differences in the analytical methods. 

Using DTT data, Crofts and colleagues [137] investigated the anatomical network efficiency changes after stroke. They proposed a measure of communicability that estimates the ease through which information can travel across a network. They found reduced communicability in stroke patients in both regions surrounding the lesions in the affected hemisphere and homologous locations in the contralesional hemisphere. They also identified regions with increased communicability in patients that could represent adaptive, plastic changes after stroke [137]. 

## 4. Intervention Effects on Connectivity in Stroke Patients

Much evidence has suggested that stroke patients will benefit significantly from rehabilitative therapies beyond spontaneous recovery of function [138, 139]. Recently, connectivity analyses have been used to investigate the intervention effects on brain connectivity [140]. The preliminary experimental studies have suggested that treatments with neural progenitor cells [9], sildenafil [95], or erythropoietin [96] induced increased FA in the ischemic lesion borderzone, which reflects a high density of axons and myelin in this region [9, 97]. In chronic stroke patients participating in a regimen of electrical stimulation targeting the paretic arm, after an 8-week therapy, these patients exhibited decreased mean diffusivity (MD) in the middle cerebellar peduncle and posterior limb of the internal capsule following treatment, which suggests that active rehabilitative therapies augmented by electrical stimulation may induce positive behavioral changes by increasing white matter tract integrity in regions specific to sensory-motor function [141]. In stroke patients with aphasia, both intense intonation-based speech therapy [50] and constraint induced language therapy [142] could induce increased fibers or white matter integrity in fiber tracts involved in language function. Acupuncture treatment may facilitate the recovery of motor function. Wu and colleagues observed the longitudinal changes of DTI measures after acupuncture treatment in rats with transient focal cerebral ischaemia [143]. They found significantly increased FA at the edge of the ischemic lesions in stroke rats with acupuncture treatment. The effect of acupuncture therapy for postponing Wallerian degeneration secondary to cerebral infarction has also been observed by DTI in stroke patients [144]. The authors found that acupuncture treatment was effective for protecting white matter integrity in stroke patients. 

The rsFC was assessed before and after a 12-week robot-aided motor rehabilitation program. The authors found that the rsFC between the ipsilesional and contralesional M1 reduced after a bout of motor rehabilitation. Greater reduction in the interhemispheric rsFC was associated with greater gains in motor function induced by the 12-week robotic therapy program. These findings suggest that greater reduction in interhemispheric rsFC in response to a bout of motor rehabilitation may predict greater efficacy of the full rehabilitation program [145]. A recent study examined the effects of upper-extremity robot-assisted rehabilitation (MANUS) versus an electroencephalography-based brain computer interface setup with motor imagery (MI EEG-BCI) and compared pretreatment and posttreatment rsFCs. The authors found that the individual gain in motor function over 12 weeks could be predicted by the rsFC changes before and after treatment. Both the motor function gain and rsFC changes were numerically higher in the MI-BCI group. Increases in rsFCs of the SMA, the contralesional and ipsilesional motor cortex, and parts of the visuospatial system with mostly association cortex regions and the cerebellum were correlated with individual upper-extremity function improvement [146]. A placebo-controlled, double-blind, and crossover design study was performed to investigate the effects of noradrenergic stimulation on the cortical motor system in hemiparetic stroke patients [147]. Effective connectivity analyses with DCM revealed that in stroke patients neural coupling with the SMA or vPMC was significantly reduced compared with healthy controls. This “hypoconnectivity” was partially normalized when patients received reboxetine (RBX), especially for the coupling between the ipsilesional SMA and M1. The data suggest that noradrenergic stimulation may help to modulate the pathologically altered motor network architecture in stroke patients, resulting in increased coupling of the ipsilesional motor areas and thereby improved motor function.

Resting-state fMRI data were analyzed using the SEM to evaluate therapy-related changes in motor network effective connectivity in stroke patients after 3 weeks of upper-extremity rehabilitation in the accelerated skill acquisition program (ASAP). The authors found that the behavioral improvement after training of the impaired upper extremity is accompanied by increased influence of the affected hemispheric PMC upon the unaffected hemispheric PMC and on the affected hemispheric M1 [148].

The relationship between behavioral recovery and interhemispheric and intrahemispheric communication has been investigated by inhibiting the contralesional M1 using1-Hz repetitive transcranial magnetic stimulation (rTMS) [149]. After inhibiting the contralesional M1 using 1-Hz repetitive transcranial magnetic stimulation (rTMS), the authors found that the motor performance of the paretic hand significantly improved. The connectivity analysis revealed that the behavioral improvements were significantly correlated with a reduction of the negative influences originating from the contralesional M1 during paretic hand movements. Concurrently, endogenous coupling between the ipsilesional SMA and M1 was significantly enhanced after rTMS was applied over the contralesional M1. The connectivity analyses suggest that both a reduction of pathological transcallosal influences and a restitution of ipsilesional effective connectivity between the SMA and M1 underlie improved motor performance [149]. 

## 5. Conclusions

A growing body of evidence from connectivity-based analyses of functional imaging data has told us that a focal stroke lesion may affect not only the lesion site but also the network to which it belongs. Thus the connectivity-based analytic methods may be more appropriate for elucidating stroke-induced impairments from a network perspective and for clarifying the mechanisms of motor recovery after stroke. Moreover, connectivity analyses are likely to be better suited to investigate the mechanisms through which therapeutic interventions may facilitate the recovery of motor function and help us to develop new intervention therapies targeting the restoration of the function of the motor network. Finally, connectivity measures may serve to monitor the process of stroke recovery and to predict the outcomes of stroke patients at an early stage. However, conflicting findings exist and the exact mechanisms leading to changes in brain connectivity after stroke remain elucidated. Therefore, longitudinal studies with large sample size employing different neuroimaging modalities covering the whole period from the superacute stage to the late chronic stage are needed to further our understanding of how different types of brain connectivity evolve after stroke and how they relate to motor deficits and clinical outcome. 
